# Pulsed field ablation vs. medical therapy for atrial fibrillation: a propensity score–matched comparison with the JoFib registry

**DOI:** 10.3389/fcvm.2026.1814114

**Published:** 2026-06-05

**Authors:** Abdalrahman Al-slaimieh, Khaled A. Abukhalaf, Nour S. Haj Ali, Jude Awad, Abdallah Al-Ani, Ayman J. Hammoudeh, Mohammad Hajjiri

**Affiliations:** 1 Department of Electrophysiology, Abdali Medical Center, Amman, Jordan; 2 Division of Vascular and Interventional Radiology, Boston Children’s Hospital, Boston, MA, United States; 3 Department of Cardiology, Istishari Hospital, Amman, Jordan

**Keywords:** atrial fibrillation, JoFib registry, Jordan, propensity score matching, pulsed field ablation

## Abstract

**Introduction:**

Pulsed field ablation (PFA) is a novel, non-thermal method for the treatment of atrial fibrillation (AF). We conducted this study, as part of one of the earliest reports originating from the MENA region, to compare the efficacy of PFA vs. medical therapy alone for the treatment of AF.

**Methods:**

A retrospective cohort study was conducted in which 62 patients who underwent PFA ablation for AF were compared with a cohort of 62 propensity score–matched patients sourced from the JoFib Registry. Rates of readmission in both arms due to AF recurrence or heart failure exacerbation driven by rapid ventricular response to AF within the period of 1 and 6 months were considered the primary study endpoints. Data were cleaned and analyzed using R 4.5.3.

**Results:**

Both cohorts were well matched in terms of age, gender, diabetes mellitus, hypertension, heart failure, left atrial diameter, CHA_2_DS_2_-VASc score, ejection fraction, and prior use of antiarrhythmic drugs. Compared with medical therapy, PFA showed significantly lower readmission rates at 1 and 6 months (3.2% and 4.8% vs. 12.9% and 17.7%; *p* < 0.05). A Kaplan–Meier analysis showed significantly higher freedom from readmission rates in the PFA cohort compared with the JoFib cohort (92% vs. 69%; log-rank *p* = 0.002).

**Conclusions:**

This early report from the MENA region aligns with the global consensus supporting PFA as a treatment for AF and lays the foundation for future larger studies to investigate PFA in this specific population.

## Introduction

Atrial fibrillation (AF) is the most common cardiac arrhythmia worldwide (1). It is estimated that the global prevalence of AF reached 52 million in 2021 based on an analysis from the Global Burden of Disease (GBD) database (2). In the United States alone, AF prevalence is projected to reach as high as 12 million by the year 2030 (3). Data sourced from the Middle East and North Africa (MENA) region show an increase of up to 800% in the age-standardized incidence rate (ASIR) of AF between the years 1990 and 2019 in some countries in the MENA region such as Qatar and the United Arab Emirates (4). These epidemiological trends carry a substantial clinical weight, as AF is associated with an increased risk of ischemic stroke, heart failure, myocardial infarction, and cognitive decline or dementia (5).

Medical therapy aiming to restore normal sinus rhythm or control the ventricular rate, along with anticoagulation to mitigate the thromboembolic risks attributed to AF, has been the mainstay of treatment for decades (6). In lower-to-middle-income countries like Jordan, medical therapy for the treatment of AF is still widely used mostly because of its cost-effectiveness (7). However, pulmonary vein isolation (PVI) using thermal and non-thermal modalities of catheter ablation is becoming widely acceptable as a safe and effective treatment for AF (8). Pulsed field ablation (PFA) is an innovative, non-thermal method of cardiac myocyte ablation that induces a safe PVI through irreversible electroporation (IRE), while sparing adjacent sensitive structures (9).

As a result, presently, AF is viewed as a disease spectrum rather than a distinct class, and there is a move toward early rhythm control using catheter ablation to improve long-term outcomes (10). However, there is often a debate about the placebo effect behind catheter ablation, which was the focus of previous large studies like the SHAM-PVI study (11). In this study, we compare one of the earliest cohorts undergoing PFA ablation for AF from the MENA region with a propensity score–matched cohort pooled from the Jordan Atrial Fibrillation (JoFib) registry to elucidate the effectiveness and safety of PFA and to further investigate the probability of the placebo effect behind catheter ablation, including PFA.

## Methodology

**Figure 1:**
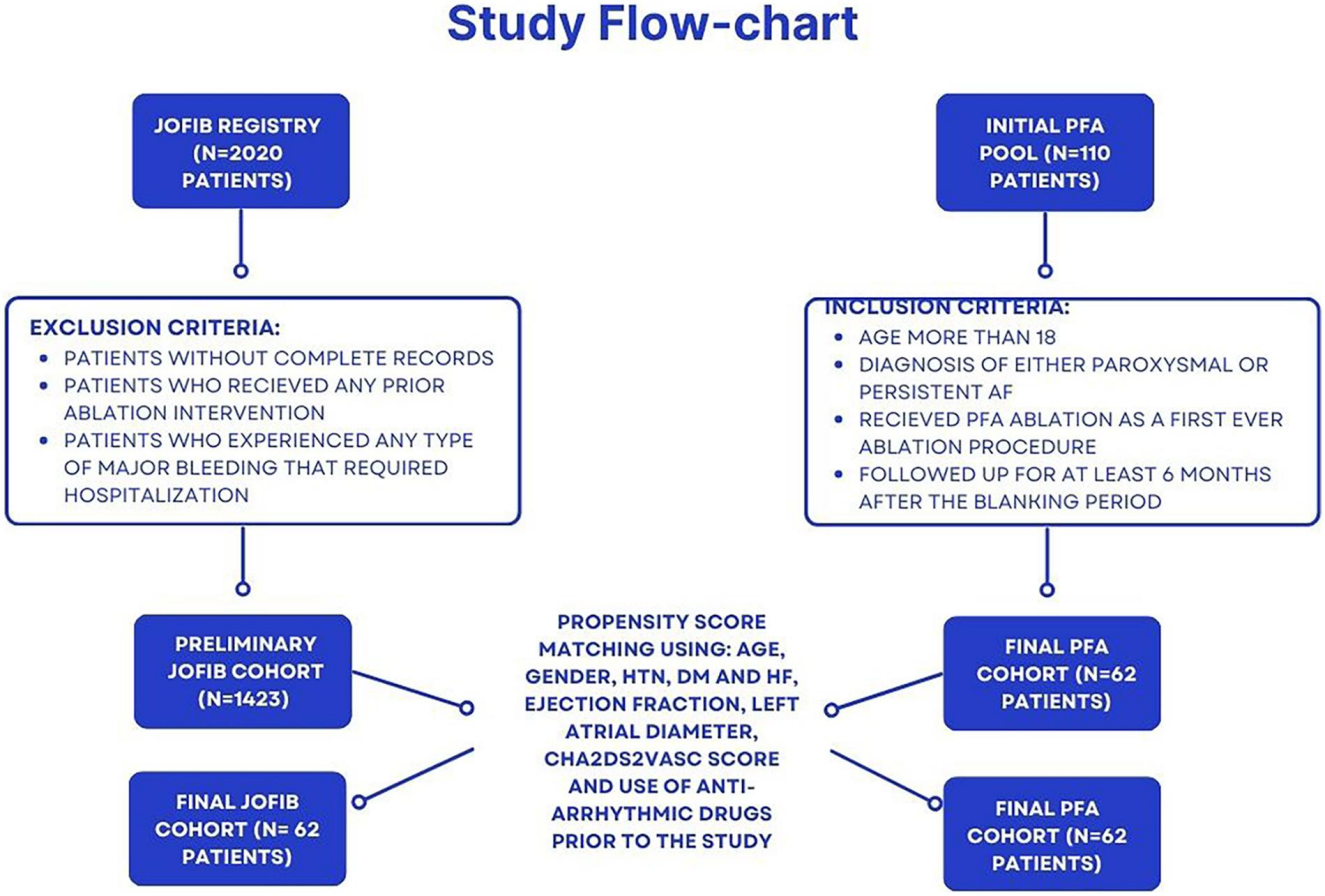
The study flow chart. PFA, pulsed field ablation; JoFib, Jordanian Atrial Fibrillation registry.

This is a retrospective cohort study approved by the Institutional Review Board (IRB) at the Abdali Medical Center. The study protocol complies with the declaration of Helsinki as updated in 2024. The need for informed consent was waived by the IRB because of the retrospective nature of the study design. Access to the JoFib Registry database was granted upon contacting the relevant authorities. The study flowchart is depicted in Figure 1.

### PFA study population and procedural characteristics

The records of patients who underwent PFA ablation for AF at a single tertiary center in Jordan between January 2025 and January 2026 were retrieved. All patients older than 18 and who received a diagnosis of either paroxysmal or persistent AF based on the 12-lead electrocardiogram (ECG) or 24 h Holter monitor and consequently underwent their first ablation procedure at this center, in which PFA was used, were included in the study. Patients who received prior catheter ablation or did not reach the 6-month follow-up mark after the index PFA procedure were excluded. Patients' charts, operation notes, ECGs, Holter monitor results, and trans-thoracic echocardiogram (TTE) findings were retrieved from their electronic charts.

All procedures were carried out by a single operator in a single facility using the Medtronic PulseSelect PFA system and three-dimensional (3D) electroanatomic mapping (EAM) under general anesthesia by a board-certified anesthesiologist and with continuous input from the TEE throughout the procedure. Ultrasound-guided femoral access was obtained in all patients followed by the introduction of the transseptal 8-French sheath and PulseSelect ablation catheter to achieve transseptal puncture (TSP) and PVI, respectively. Patients were admitted to the telemetry unit after the procedure for a 24 -h observation period, and a 12-lead ECG was performed on all patients prior to discharge. Clinical follow-up was conducted on all patients at the following time points: 1 week, 3 months, and 6 months. Holter monitoring was performed at 3 months after the procedure for all patients regardless of the symptoms to confirm normal sinus rhythm.

Among the matched PFA cohort, the mean procedural time was 72 ± 9.1 min, while the mean fluoroscopy time was 18 ± 1.2 min. 3D EAM was performed in 14 patients (22.6%).

### JoFib registry cohort

The JoFib registry was a multicenter observational registry that enrolled patients with an established diagnosis of either paroxysmal of persistent AF based on the 12-lead ECG, a rhythm strip lasting more than 30 s, more than one episode of AF on ambulatory ECG, or a past diagnosis by a treating cardiologist. The registry included detailed descriptions of patients' demographics, comorbid conditions, medication history, TTE findings, and hospitalization characteristics from May 2019 to October 2020.

### Propensity score matching technique

A 1-to-1 propensity score matching technique was adopted to rectify the imbalance of covariates between the PFA and the JoFib cohorts. The technique involved calculating the optimal standardized mean differences using the “optimal” method. Distances between covariates were measured using a general linear model (GLM). A caliper width equal to 0.2 of the standard deviation of the logit of the propensity score was used (12, 13). Both the PFA and the JoFib cohorts were matched in terms of the following variables: age, biological sex, hypertension (HTN), diabetes mellitus (DM), heart failure (HF), CHA_2_DS_2_-VASc score, ejection fraction (EF), left atrial diameter (LAD) as measured in the parasternal long-axis view using a two-dimensional transthoracic echocardiograph, and the use of antiarrhythmic drugs (AADs) including class I and class III. A love plot comparing the balance diagnostics before and after matching is available in Supplementary Figure S1.

### Definition of the outcome and statistical analysis

Data were cleaned and analyzed using R (4.5.3). The data were presented using descriptive statistics and survival analysis using a Kaplan–Meier curve. Continuous variables were presented as mean ± standard deviation (SD), while categorical variables were presented as frequencies with associated percentage values. Differences between categorical variables were examined using the chi-square test. Differences between continuous variables were examined using an independent sample *t*-test. The primary study endpoint was considered the rate of readmissions for AF recurrence or HF exacerbation driven by rapid ventricular response due to AF that required hospitalization for intravenous diuresis with or without the need for supplemental oxygen within the 1- and 6-month follow-up period in both cohorts. A Kaplan–Meier survival analysis was performed to estimate the 6-month freedom from hospital readmission rates for the primary study endpoint in both cohorts, and data were presented as cumulative survival percentage with 95% confidence interval (CI). Differences in freedom from hospital readmissions between the included cohorts were examined using the log-rank test. A *p*-value of less than 0.05 was considered statistically significant.

## Results

Table 1 shows the matched baseline characteristics between the PFA and the JoFib cohorts. The two cohorts were balanced in terms of age (PFA: 60.4 ± 16.3 vs. JoFib: 61.3 ± 12.9; *p* = 0.72), gender (*p* = 0.86), HTN (*p* = 0.35), DM (*p* = 0.85), HF (*p* = 1.00), CHA_2_DS_2_-VASc score (PFA: 2.2 ± 1.8 vs. JoFib: 2.5 ± 1.4; *p* = 0.25), EF (PFA: 55.3 ± 9% vs. JoFib: 55.9 ± 12.1%; *p* = 0.76), LAD (PFA: 3.9 ± 0.7 vs. JoFib: 3.9 ± 0.8; *p* = 0.72), and prior use of AADs (*p* = 0.57). Furthermore, there were no statistically significant differences between the two cohorts in terms of usage distributions of *β*-blockers (*p* = 0.22), renin–angiotensin–aldosterone system inhibitors (*p* = 0.28), lipid-lowering agents (*p* = 0.14), and diuretics (*p* = 0.18). However, the PFA cohort was significantly more likely to smoke (*p* = 0.03) and use calcium channel blockers (*p* = 0.009).

**Table 1:** Basic clinical, social, and medication profiles of the included cohorts.

Characteristic	PFA (*n* = 62)	JoFib (*n* = 62)	*p*-Value^a^
Age (mean ± SD)	60.35 ± 16.3	61.31 ± 12.9	0.72
Male sex, *n* (%)	33 (53.2)	32 (51.6)	0.86
Hypertension, *n* (%)	37 (59.7)	42 (67.7)	0.35
Diabetes mellitus, *n* (%)	22 (35.5)	23 (37.1)	0.85
Heart failure, *n* (%)	11 (17.7)	11 (17.7)	1.00
Use of AADs, *n* (%)	22 (35.5)	19 (30.6)	0.57
Ejection Fraction (mean ± SD)	55.3 ± 9%	55.9 ± 12.1%	0.76
CHA_2_DS_2_-VASc (mean ± SD)	2.2 ± 1.8	2.5 ± 1.4	0.25
Left atrial diameter (mean ± SD)	3.9 ± 0.7	3.9 ± 0.8	0.72
Smoking, *n* (%)	26 (41.9)	14 (22.6)	0.03
*β*-Blockers, *n* (%)	55 (88.7)	49 (79.0)	0.22
Calcium channel Blockers, *n* (%)	17 (27.4)	5 (8.1)	0.009
RAAS inhibitors, *n* (%)	31 (50.0)	24 (38.7)	0.28
Diuretics, *n* (%)	24 (38.7)	16 (25.8)	0.18
Lipid-lowering agents, *n* (%)	27 (43.5)	18 (29.0)	0.14

a Differences between categorical variables were examined using the chi-square test. Differences between continuous variables were examined using an independent sample *t*-test. PFA, pulsed field ablation; RAAS, renin–angiotensin–aldosterone system.

**Figure 2:**
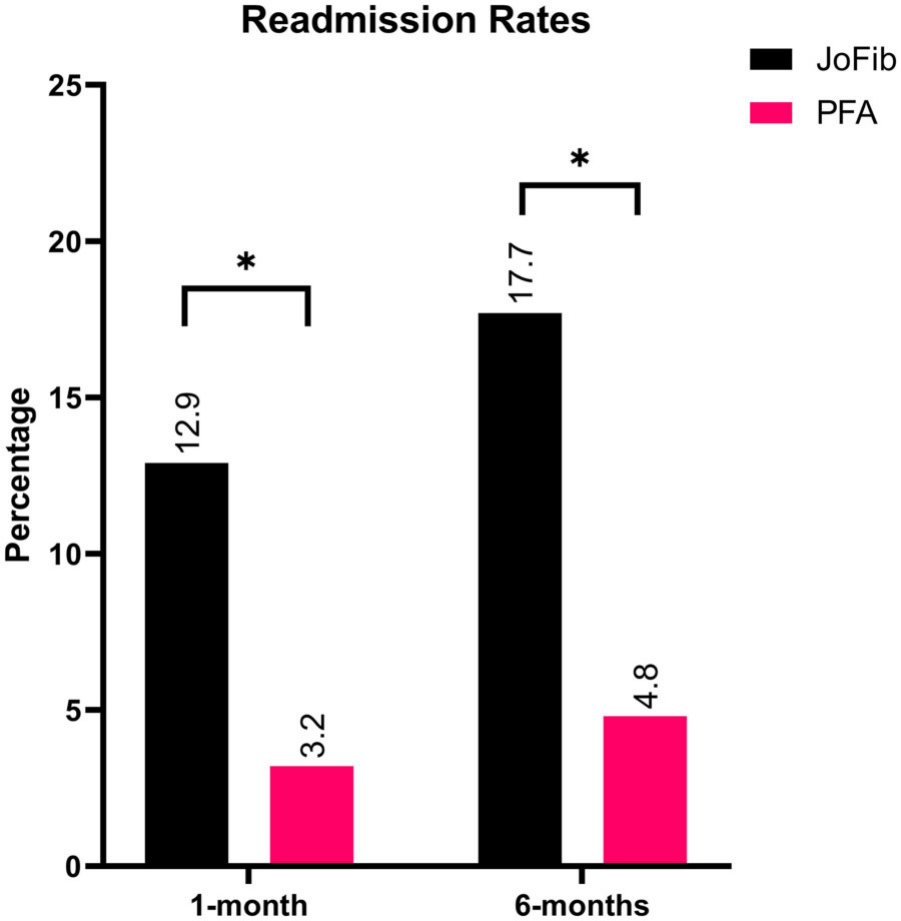
Rates of readmissions within the two cohorts at 1- and 6-month follow-up periods. *Statistically significant difference at *p*-value < 0.05.

After 1 month, the study's primary endpoint was observed in 2 (3.2%) of the study participants in the PFA cohort, while it was observed in 8 (12.9%) participants in the JoFib cohort. The distribution of readmission at 1 month was significantly different between the PFA and the JoFib cohorts (*p* = 0.048). After 6 months of follow-up, a total of 3 (4.8%) patients in the PFA cohort and 11 (17.7%) in the JoFib cohort experienced the primary study endpoint (*p* = 0.023) (Figure 2). In the PFA group, the only indication for readmission that was reported was AF recurrence. Among the 8 patients readmitted at 1 month in the JoFib cohort, 37.5% (*n* = 3) of readmissions were attributed to AF recurrence and 62.5% (*n* = 5) of readmissions were due to HF exacerbation driven by AF. At 6 months, among the 11 patients readmitted in the JoFib cohort, 45.5% (*n* = 5) were attributed to AF recurrence and 54.5% (*n* = 6) were due to HF exacerbation driven by AF.

**Figure 3:**
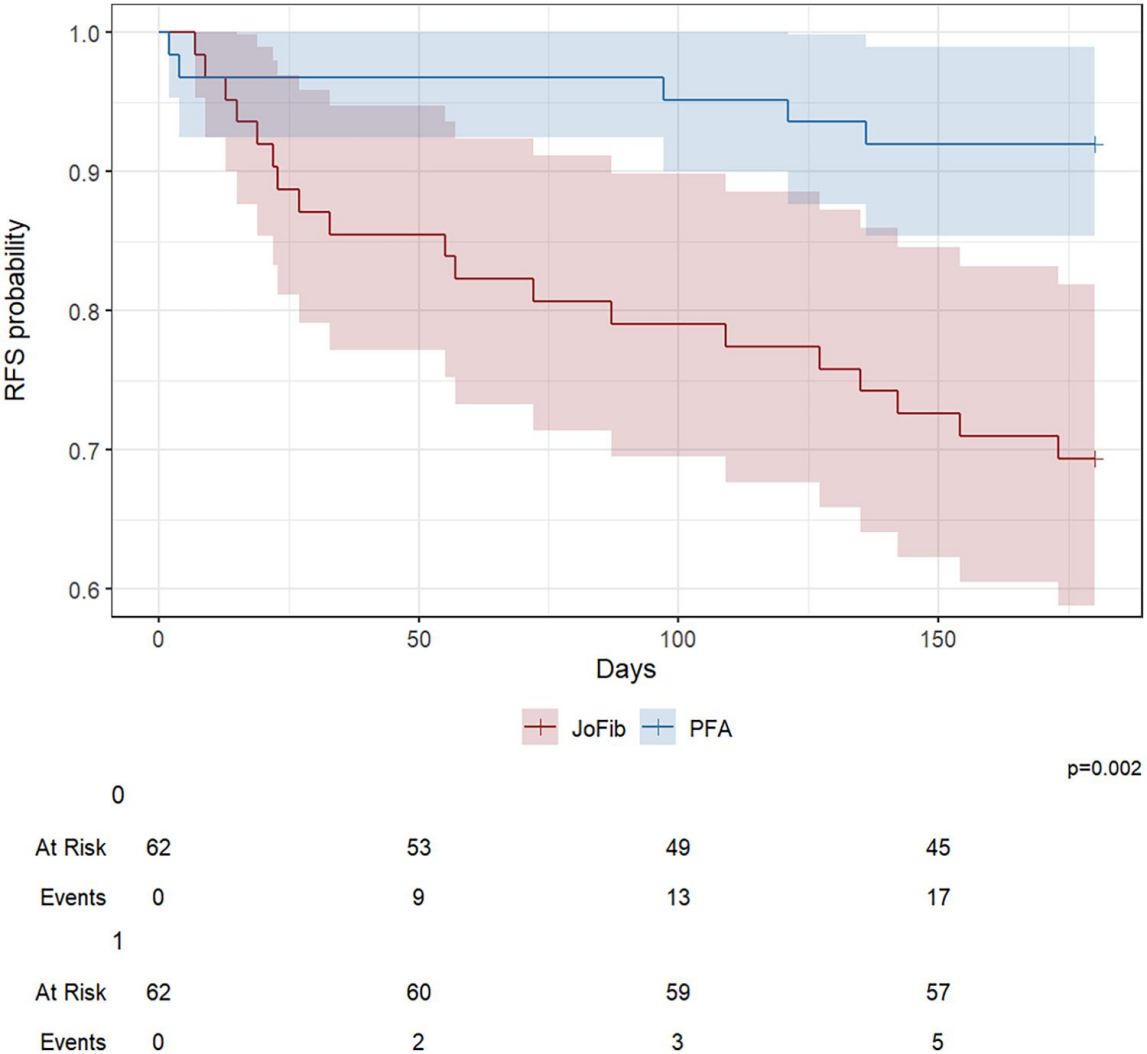
A Kaplan–Meier curve showing the cumulative AF-recurrence survival proportion in the two included cohorts.

A Kaplan–Meier survival analysis demonstrated a clinically significant difference between the two groups in terms of 6-month freedom from rehospitalization. Specifically, the PFA group demonstrated a 92% (95% CI: 85%–99%) cumulative readmission-free survival rate, while the JoFib cohort exhibited a 69% (95% CI: 59%–82%) cumulative readmission-free survival rate (log-rank *p* = 0.002) (Figure 3).

## Discussion

In present times, early catheter ablation is being increasingly used as a method of rhythm control. One trial showed that early rhythm control using antiarrhythmic medications or catheter ablation was associated with a lower risk of adverse cardiovascular outcomes compared with usual care (i.e., patients treated with rate control therapy only for symptom mitigation) (14). Furthermore, it appears that catheter ablation is linked to better rhythm control in comparison with antiarrhythmic medications; this was first proved in the CABANA trial (15). Although the thermal modes of ablation (e.g., radiofrequency and cryoablation) have been performed with relative consistency over the years, they are limited by their potential to cause collateral tissue damage and long application times (9).

In this context, PFA presents itself as an innovative technology for AF ablation that is both safe and effective in the treatment of AF. This is attributed to the inherent property of the non-thermal energy utilized in PFA (i.e., IRE) to achieve PVI without thermally induced complications (9). The safety profile of PFA is further enhanced by the shorter procedural time that distinguishes PFA from its thermal counterpart (16). Preclinical evidence shows that cardiac cells are preferentially affected by PFA compared with pulmonary, esophageal, or myelinated nerve cells (9), as intentional overablation of these tissues has failed to cause any substantial damage (17). Interestingly, it is only hypothesized but not confirmed that the efficacy of PFA is associated with its ability to induce necrosis to the periarterial ganglionated plexi (18, 19).

Our study further underscores the efficacy profile of PFA, highlighted as significantly lower rates of readmission-free survival at both the 1- and 6-month follow-up time points compared with the JoFib cohort who received only the standard of care without ablation. The safety and efficacy profiles of PFA are often attributed to a number of factors. The literature shows that the use of circular or pentaspline catheters within PFA systems leads to the delivery of faster, circumferential, and near-instantaneous energy capable of isolating the pulmonary vein, in turn, leading to short left atrial dwelling times (20). In addition, delivering the system's energy in the form of IRE helps induce cell apoptosis by increasing cell membrane permeability, without triggering excessive inflammatory responses (9). This translates into a longer safety window associated with PFA. In our PFA cohort, the average procedure time was 72 ± 9.1 min, which is significantly lower than that of traditional methods (21, 22). It appears that both the duration of the procedure and ablation are predictors of AF recurrence (23, 24). A higher operative duration will increase the likelihood of thermal injury, thus sustaining transient inflammatory responses that may predispose to early interventional failure.

The current Jordanian health infrastructure is dominated by public, government-funded institutions. This predisposes the healthcare landscape to a high degree of rigidity in terms of accepting new standards of care. At the clinical, operational, and economic levels, PFA offered a cheap, efficient, and effective alternative to traditional ablation technologies and antiarrhythmic drugs in the past (25, 26). Yet, only a single Jordanian center managed to adopt the technology and offer its advantages to patients. It appears that the high price tag of PFA procedures, driven by equipment and training (27), may act as a barrier to their use in Jordan. In addition, the lack of competition, even at the level of the private sector, may lead to the stagnation of PFA pricing. Therefore, the commitment to the initial capital required to provide PFA technology or any other new ablation treatments will mostly remain a barrier to any long-term cost reductions in the form of lower disease burden.

Although PFA is now considered one of the latest fads in the world of electrophysiology, the technology does have its fair share of limitations and pitfalls. Up until this moment, the optimal settings, dosage, and technique to achieve a precise, durable, and transmural ablation with minimal myocardial injury are yet to be defined (28). Moreover, although only two PFA systems are approved, the settings, validation, indications, and results of each system significantly differ from one another and are not transferable (29). Also, the MANIFEST—17 K multicentric investigation demonstrated rare, yet extremely morbid, adverse effects (30), which include coronary spasms, proximity-related vasospasm, generalized vasospasm, and hemolysis in the setting for acute renal failure. The PEACE-AF (NCT07064616) also reported asymptomatic cerebral emboli after PFA (31). We suggest that widespread adoption of such technology may be feasible only after elucidating the significance of these adverse effects through long-term data and robust randomized controlled trials.

To the best of our knowledge, this study is considered one of the earliest reports originating from the MENA region to explore the effectiveness and safety of PFA compared with standard medical therapy alone. Furthermore, our results align with the growing global consensus that supports the early use of PFA intervention for rhythm control in both paroxysmal or persistent AF. Despite the relatively modest sample size of the study, the application of PSM enhanced the comparability of both cohorts and mitigated the impact of covariates, thereby enhancing the internal validity of our comparative outcomes.

However, the results of this study should be interpreted in the context of certain important limitations. The 6-month follow-up period precludes definitive conclusions on the long-term safety profile of PFA; thus, future projects with extended longitudinal data are essential. Also, the retrospective, single-center design through which the PFA cohort received their treatment, necessitated by the current limited availability of PFA systems in Jordan, limits the generalizability of our findings. Moreover, despite the availability of a well-balanced set of cohorts to compare the traditional ablation technique with PFA, not all confounders of AF or its treatment were included in the study because of limitations within the sources of data (e.g., adherence to medications). Finally, because of the limited number of outcome distributions within a modest sample size, a rigorous statistical examination of outcome data was hampered, and uncertainty was projected through wide confidence intervals. Nevertheless, this study establishes a vital foundation for future, large-scale, multicenter investigations to evaluate the safety, effectiveness, and cost-effectiveness of PFA within the unique healthcare structure of the MENA region.

## Data Availability

The raw data supporting the conclusions of this article will be made available by the authors, without undue reservation.
