# Supplementary figures and images for: Pulsed field ablation vs. medical therapy for atrial fibrillation: a propensity score–matched comparison with the JoFib registry
Source: Front Cardiovasc Med. 2026 Jun 5;13:1814114. doi: 10.3389/fcvm.2026.1814114 (PMC13278897; doi:10.3389/fcvm.2026.1814114)

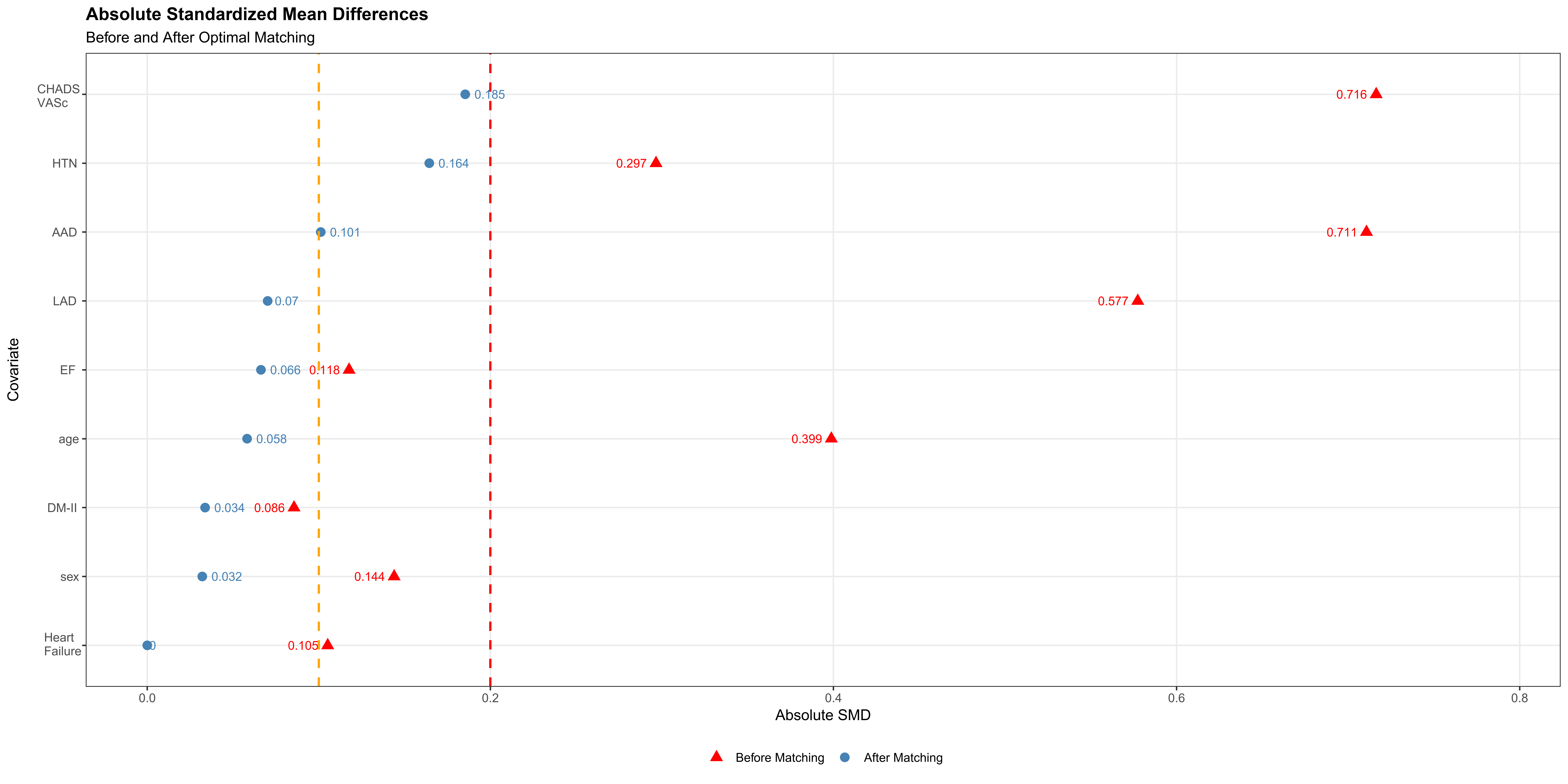

Supplement: Supplementary file 1 [file Image1.tif]
